# Biomechanics of raptorial dorsiflexion and tensile material properties of the m*.* tibialis cranialis tendon in the hindlimbs of hawks and owls

**DOI:** 10.1242/jeb.251052

**Published:** 2025-12-10

**Authors:** Alexander D. Clark, Linnea L. Lungstrom, Samantha J. Clark, Mark W. Westneat

**Affiliations:** ^1^ Committee on Evolutionary Biology, University of Chicago, Chicago, IL 60637, USA; ^2^ Field Museum of Natural History, 1400 S Dusable Lake Shore Drive, Chicago, IL 60605, USA; ^3^ Miami University, College of Arts and Science, Oxford, OH 45056, USA; ^4^ Department of Organismal Biology and Anatomy, University of Chicago, Chicago, IL 60637, USA

**Keywords:** Organismal torque, Birds of prey, Muscle efficiency, Bird anatomy, Ecology

## Abstract

Skeletal morphology and tendon properties reveal the underlying systems that facilitate movement and, ultimately, behavior in animals. Of all terrestrial vertebrates, birds are the most diverse in terms of species. Birds often interact with their prey via the hindlimbs, making these portions of the body rich in ecomorphological information. The m. tibialis cranialis is the primary dorsiflexor of the ankle, and in raptorial birds, this muscle critically aids in both prey acquisition and transport. Here, we assessed the biomechanical implications of tarsometatarsal morphology and the tensile properties of the m. tibialis cranialis tendon among accipitrids and strigids to assess potential differences in prey acquisition strategies and load capacities. Examination of tarsometatarsal morphology reveals significantly more robust and much more distally positioned insertion points for the m. tibialis cranialis in strigids, resulting in comparatively increased mechanical advantage. Though acquisition strategies among focal raptorial birds differed, results of tensile properties indicate no significant differences in mass-specific load-carrying capabilities. However, results suggest that strigids only require approximately half the torque required by accipitrids to lift a given resistance, even when adjusting for mass categories. The elastic modulus of m. tibialis cranialis tendons was low across examined birds, ranging from 17.2 to 128 MPa. Our central conclusions are that tarsometatarsal morphology indicates differences in mechanical lever arm advantage between the two groups, and that hindlimb tendons of arboreal birds of prey are relatively elastic, with significantly lower moduli than those of cursorial birds and mammals, suggesting functional roles in shock absorption and strain energy storage during lifting and transport of prey.

## Teaser

**Summary:** Skeletal morphology and tendon tensile properties of raptorial bird hindlimbs yield insight into prey capture capabilities, with surprisingly elastic tendon modulus values compared with mammals and terrestrial birds.

## INTRODUCTION

Among birds of prey, variations in prey acquisition strategies are often reflected in the morphologies of the hindlimbs, particularly concerning grasping and grip strength (Fowler et al., 2009; Goslow, 1967, 1972; Moreno and Carrascal, 1993; Zeffer and Norberg, 2003; Ward et al., 2002). Species within the families Accipitridae (eagles, hawks and kites) and Strigidae (true owls), though all raptorial, have unique skeletal disparities that signal ecomorphological differences in how they contact, subdue and transport prey (Madan et al., 2017; Volkov, 2004; Ward et al., 2002). For example, accipitrids exhibit proportionally longer legs than similarly sized strigids, in part owing to proportionally longer tarsometatarsi relative to other bones of the hindlimb. These skeletal differences within the hindlimb result in significantly different biomechanical forces and resulting mechanical advantage values (Allen et al., 2021; Clark et al., 2024; Kambic, et al., 2017; Lin et al., 2024; Volkov, 2004; Ward et al., 2002). These differences, in turn, also affect the relative locations of muscle insertions, potential tendon widths and, subsequently, the amount of strain that can be applied (Volkov, 2004; Ward et al., 2002). Indeed, previous studies have shown that strigids tend to have relatively increased potential grip strength compared with accipitrids, attributable to both a larger m. flexor digitorum (Ward et al., 2002) and wider plantar tendons throughout the pedal digits (Volkov, 2004). Although these soft-tissue data are informative, assessment of hindlimb tendon tensile properties, which can greatly inform on *in vivo* use, have yet to be thoroughly assessed.

The relationship between tendon tensile strength and behavior is well known among mammals, which have received proportionally greater attention (Bennett et al., 1986; Mossor et al., 2020; Pollock and Shadwick, 1994; Summers and Koob, 2002; Wells, 1965). Assessment of tensile strength among wild birds is more limited, with the wild turkey (*Meleagris gallopavo*) being the most represented species (Bennett and Stafford, 1988; Kadar et al., 2017; Landis et al., 1995; Matson et al., 2012). *Meleagris gallopavo* undergoes a stage of tendinous precalcification early in ontogeny to then full calcification of the hindlimb tendons, greatly affecting their relative stiffness and load capacity throughout their growth (Bennett and Stafford, 1988; Kadar et al., 2017; Leonard et al., 1980). The m. tibialis cranialis tendon in particular in *M. gallopavo* can withstand comparatively high stresses, being second only to the m. flexor hallicus longus (Matson et al., 2012). These dorsal and plantar tendons facilitate stability and flexion of the ankle, and likely serve a critical role in this large-bodied terrestrial-locomoting bird (Landis et al., 1995; Matson et al., 2012).

Examination of the interplay between skeletal variation and tensile strength of soft tissues of the hindlimb in accipitrids and strigids is largely unexplored (Olmos et al., 1993; Volkov, 2004; Ward et al., 2002). Though pedal flexion has significant implications for prey acquisition, so too does dorsiflexion of the ankle, which in raptorial birds is crucial during pinning down, grasping and transporting prey. Dorsiflexion of the ankle is facilitated by contraction of the m. tibialis cranialis, which is located on the dorsal face of the tibiotarsus and inserts on the dorsal face of the tarsometatarus.

Here, our central goal was to assess the biomechanical and ecological implications of both the morphology of the tarsometatarsus and the tendinous tensile properties of the m*.* tibialis cranialis and their role in the raptorial tarsometatarsal gripping and lifting lever mechanism in six birds of prey – three accipitrids and three strigids – with each species representing a unique size category, prey preference and subsequent prey acquisition strategy. We hypothesized that owls would exhibit higher load-bearing capabilities of the m. tibialis cranialis tendon relative to hawks. Though previous studies have found the belly of this muscle to have similar cross-sectional area between members of these families, we based this prediction on two primary factors: disparities in foraging strategies and previous evidence suggesting that owls have greater grip strength relative to hawks (Ward et al., 2002). Strigids, unlike accipitrids, have a greater propensity to constrict and crush prey items, often with greater proportional masses to their own (del Hoyo et al., 1999; Madan et al., 2017; Ward et al., 2002).

Outcomes of these assessments may biomechanically explain how the hindlimbs of these six different species of raptorial birds facilitate prey acquisition and their differences in prey-carrying thresholds. Additionally, as these species are arboreal, we predict different stress–strain curves and elastic modulus values than those of terrestrial birds such as *M. gallopavo*. Although these raptors perform sudden high-force behaviors with their hindlimbs, we predict that the elevated stiffness and resilience of *M. gallopavo* tendons used for terrestrial walking and running (similar to mammals) will not be present in these raptorial taxa.

A second broader aim of this study was to expand the survey of tarsometatarsal morphology and lever dimensions to a wider set of strigid and accipitrid species, including extinct species assigned to these groups. Assessing the underlying skeletal and muscular systems that facilitate behaviors in extant birds of prey opens the possibility to ask similar questions concerning atypically large or unique morphology-bearing extinct members within the same family.

## MATERIALS AND METHODS

### Comparative tarsometatarsus anatomy

**Fig. 1.**
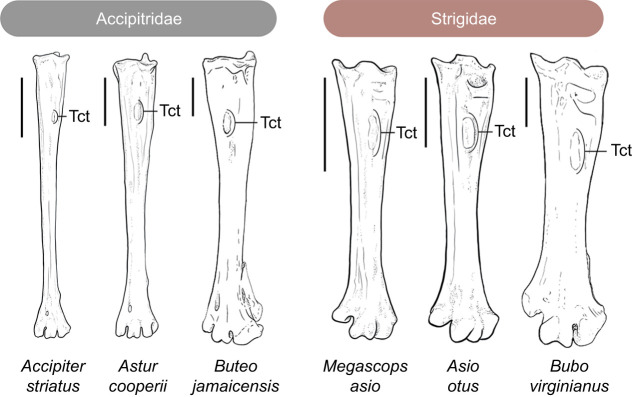
**Morphologies of the tarsometatarus among sampled species.** All show the right tarsometatarsus in dorsal aspect. Note the more elongate morphology of accipitrids compared with strigids, and the proportional distal position of the m. tibialis cranialis tubercle (Tct) in strigids. The relative position of this tubercle varies within families as well. Tarsometatarsi not shown to scale. Scale bar for each tarsometatarsus equals 10 mm.

Comparative measurements of the tarsometatarsus and the insertion points of the m. tibialis cranialis were collected from individuals from the Field Museum (Chicago, IL, USA) of each focal species – the accipitrids *Accipiter striatus* Vieillot 1808 (sharp-shinned hawk), *Astur cooperii* (Bonaparte 1828) (Cooper's hawk) and *Buteo jamaicensis* (Gmelin 1788) (red-tailed hawk), and the strigids *Megascops asio* (Linnaeus 1758) (eastern screech-owl), *Asio otus* (Linnaeus 1758) (long-eared owl) and *Bubo virginianus* (Gmelin 1788) (great horned owl) (Fig. 1). Falconiforms were excluded owing to their more distant phylogenetic relatedness compared with strigiforms and accipitriforms and their specialized method of terminating prey using the tomial tooth, facilitating cervical dislocation (Prum et al., 2015; Sustaita, 2008). Accipitrid and strigid species were chosen based on their availability, differences in typical prey and size categories. Mass categories were as follows: heavy, *B. virginianus* (900–1800 g) and *B. jamaicensis* (680–1600 g); medium, *A. cooperii* (275–667 g) and *A. otus* (204–409 g); and light, *A. striatus* (95–200 g) and *M. asio* (163–263 g) (Artuso et al., 2022; Bildstein et al., 2020; Marks et al., 2020; Preston and Beane, 2020; Ritchison et al., 2020; Rosenfield et al., 2020).

To account for variation among individuals and between the sexes, skeletal measurements were gathered from 10 specimens for each species, drawing from both male and female specimens (Table S1). Osteologically mature individuals were used for this analysis as indicated by either label or level of observable ossification throughout the skeleton. For the tarsometatarsus, length (as a measure of proximodistal distance) and width (as a measure of mediolateral distance) at the midpoint were recorded using digital calipers (OriginCal1P54). Similarly, aspect ratios of the tarsometatarsus were calculated by dividing the total length by the width at the midpoint. The m. tibialis cranialis tubercle was denoted by cranial projection off the tarsometatarsus. M*.* tibialis cranialis tubercle distance was measured from the proximal margin of the tarsometatarsus to the center of the m*.* tibialis cranialis tubercle. Proportional length of the tubercle's face was relative to the total length of the tarsometarsus. The skeletal measurements from all 10 individuals for each species were averaged to create a representative individual for each species. To assess levels of significance between skeletal metrics and required muscle output force to lift prey items between the two independent groups, we used Mann–Whitney *U*-tests, as Shapiro–Wilk normality tests and Bartlett's tests showed our data to be non-normal and have unequal variances, respectively.

To assess similar tarsometatarsal morphology in extinct taxa, we used either photos from the literature or 3D scans. Two extinct accipitrids were assessed: *Hieraaetus moore* (accipitrid from the Pliocene extending into the Pleistocene, ∼5–0.05 Ma) (Mather et al., 2023) and *Dynatoaetus gaffe* (accipitrid from the Pleistocene extending into the Holocene, ∼2 Ma to <600 years ago) (Bunce et al., 2005). Similarly, two extinct strigiforms (assignment of family Strigidae is tentative) were assessed: *Mosurnia diurna* (a strigiform from the Miocene, ∼9.5–6 Ma) (Li et al., 2022) and *Minerva leptosteus* (a protostrigid strigiform from the Eocene, ∼49 Ma) (Mayr et al., 2020) (Table S2).

Lastly, to create a broader tarsometatarsus dataset for these extinct species to be compared with, we gathered anatomical metrics from an additional 34 accipitrid and 28 strigid species (Table S2). For the broader sample, we used parametric statistical tests to compare the two independent groups. Because species in these two groups are aggregated into sub-lineages, we ran a phylogenetic, RRPP corrected model (Adams and Collyer, 2018). The large time-calibrated synthesis tree of all birds by McTavish et al. (2025) (v. 1.5) was pruned to the hawk–owl region of the tree using Mesquite 4.0 (https://www.mesquiteproject.org) and further pruned to our set of 62 species using drop.tip in the R package ape (Paradis and Schliep, 2019) (see https://figshare.com/articles/dataset/Raptor_Tendon_Supporting_Files/30556223). We computed phylogenetic signal (Pagel's lambda) using the phytools function phylosig (Revell, 2024). We extracted the variance–covariance matrix from this pruned phylogeny using the vcv.phylo function in ape (Paradis and Schliep, 2019). Using this as the covariance matrix, we performed phylogenetic ANOVAs for individual traits and phylogenetic MANOVAs for two sets, one consisting of all anatomical traits and one consisting of all functional traits, using the function lm.rrpp in the package RRPP (Collyer and Adams, 2018). Anatomical nomenclature primarily follows Baumel and Witmer (1993) using the English equivalents of the Latin terminology.

### Muscle force and hindlimb torque of the m*.* tibialis cranialis

**Fig. 2.**
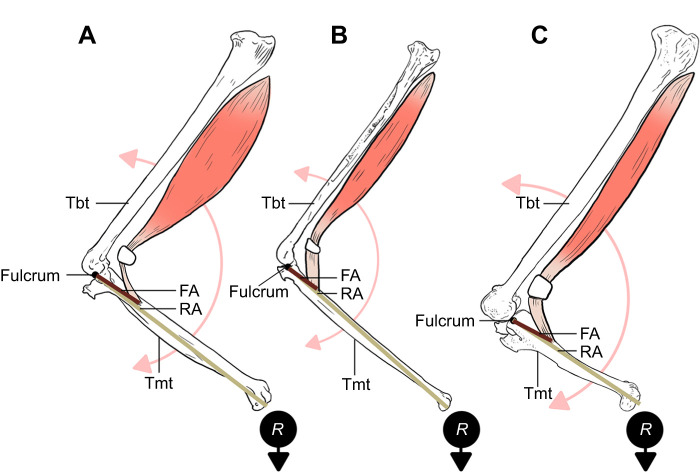
**The left leg as a third-order lever in three species.** All shown in medial aspect. The muscle belly and tendinous portion of the m. tibialis cranialis are shown with all other muscle groups removed. (A) *Buteo jamaicensis*, (B) *Accipiter striatus*, (C) *Bubo virginianus*. Areas of biomechanical significance are labeled. FA, distance from the fulcrum to the level of muscle insertion (maroon line); RA, distance from the fulcrum to the area of resistance (i.e. the weight) (gold line); *R*, hypothetical weight (i.e. resistance) of the object in the pes; tbt, tibiotarsus; tmt, tarsometatarsus. The light red line and arrows denote the area of rotation around the ankle joint (i.e. the fulcrum). Figures are not to scale.

Using measurements of the tarsometatarsus, we created third-class lever models to calculate the force and torque (rotational movement at the ankle joint) of the m. tibialis cranialis required to lift prey in each species (Fig. 2). To compute muscle force required to lift a particular mass in each species, we used the standard lever equation:
(1)

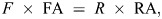
where *F* equals the amount of muscle force, FA (force arm) equals the distance from the fulcrum (i.e. joint) to the point of muscle insertion (quantified as the center of the m. tibialis cranialis tubercle), *R* equals the theoretical resistance force the hypothetical prey exerts on the pes, which is calculated by multiplying the mass of the prey by the acceleration due to gravity, hereafter referred to as ‘resistance of prey’ (RP=*R*×9.81 m s^−2^), and RA (resistance arm) equals the distance from the fulcrum to the area of resistance (i.e. captured prey). Therefore, solving for required muscle force, we used the revised equation:
(2)

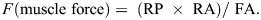
To calculate torque to lift a given prey item, we therefore used the equation (Fig. 2):
(3)

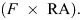
Lastly, to compare the third-class lever systems among the avian hindlimbs, we computed the mechanical advantage (MA) as the inlever FA divided by the outlever RA.

To assess required muscle forces and torque among species and prey masses, we used three prey item scenarios: (1) eastern chipmunk (*Tamias striatus*; ∼140 g), (2) Virginia opossum (*Didelphus virginianis*; ∼3600 g) (https://animaldiversity.org) and (3) the upper-end spectrum of prey known to be taken for each bird of prey species based on species' profiles in the Birds of the World database (Billerman et al., 2022). Though larger prey items are functionally impossible for several of the birds of prey sampled, the formulae will still yield the required muscle force and torque required to lift the prey item and thus allow for comparison (i.e. resulting values reveal the mechanical forces required of the limb and muscle rather than what the taxon may physically be capable of producing).

### Assessing tensile material properties of the m*.* tibialis cranialis tendon

**Fig. 3.**
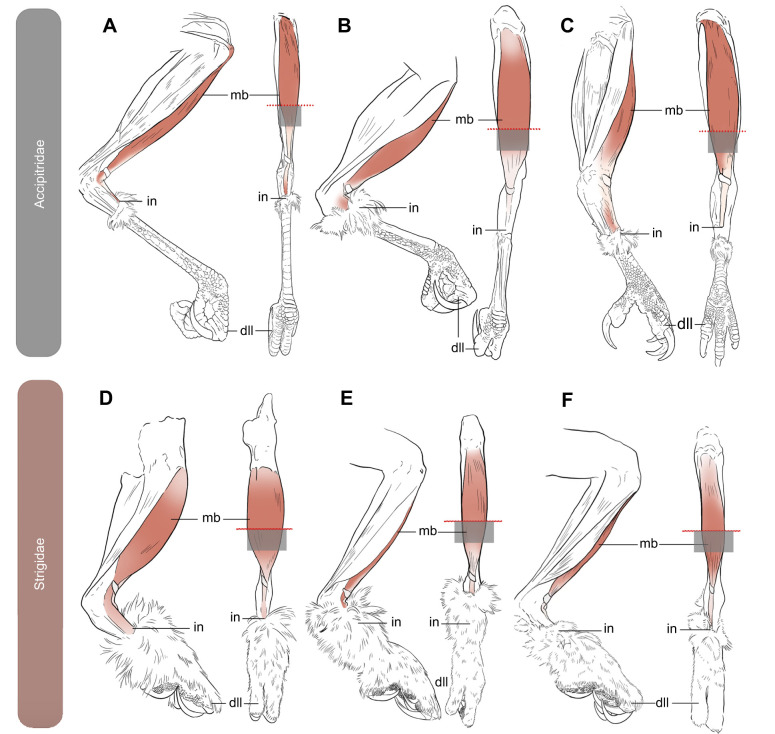
**Line drawings of the left hindlimb in medial and dorsal aspect of six raptorial birds.** Accipitrids (A) *Accipiter striatus*, (B) *Astur cooperii* and (C) *Buteo jamaicensis*, and strigids (D) *Megascops asio*, (E) *Asio otus* and (F) *Bubo virginianus*. Colored muscle represents the tibialis cranialis. Red coloration changing to ivory represents the transition from predominantly muscled area to the tendinous portion. Dotted red lines indicate the approximate location of where the muscle was cut and separated from the leg. The gray box underneath each dotted red line represents the area of clamping within the tensometer. Figures are scaled to the same height. dII, digit II; in, tendon insertion; mb, muscle belly of the tibialis cranialis.

To assess the tensile properties of the m*.* tibialis cranialis among the six extant species, deceased individuals were used for experimentation and data collection (Fig. 3). Individuals were collected under a Field Museum salvage permit (permit no. MB675461). Birds were collected either from window strike mortalities, death in captivity or death during rehabilitation. Individuals were frozen for less than 1 year and then thawed for material properties testing. All species were aged as adults by plumage. Each species had the m*.* tibialis cranialis tendon tested on both legs (*n*=2 per species).

**Fig. 4.**
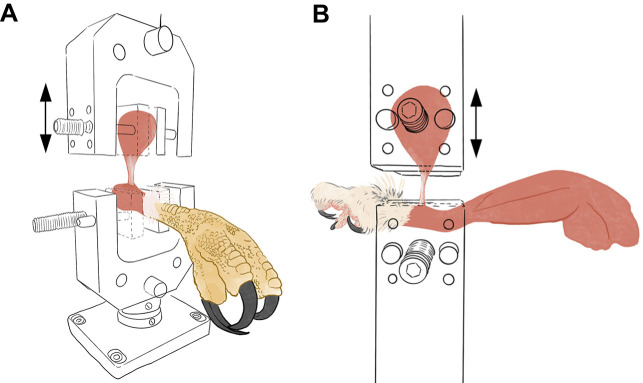
**Line drawings of the tensometer clamp methodology.** (A) *Buteo jamaicensis* and (B) *Megascops asio* serve as examples. Muscle bellies were placed in the upper clamp, whereas the tarsometatarsus was fixed into the lower clamp. Tensometer parameters were programmed to only move the upper clamp (indicated by the arrows). Specimens not shown to scale.

Each leg was loaded into a tensometer (model ESM303 – Mark 10 Series 5, 100 N gauge) using two clamps (Fig. 4). The lower clamp secured the tarsometatarsus with the insertion point of the m*.* tibialis cranialis tendon centered. The m. tibialis cranialis was cut approximately 75% distally down the muscle belly, proximal to the tendinous portion. The muscle belly was placed slightly above the upper clamp and wrapped in paper towel to prevent slippage during testing. This setup ensured that the muscle–tendon junction and the tendinous portion of the m. tibialis cranialis were the only sections exposed during increment extension testing.

Tensometer parameters were set to raise the upper clamp in a tensile test. After starting tension was applied, resting length of the m*.* tibialis cranialis tendon was measured from the insertion point to the muscular-to-tendinous transition. This resting length determined the 2% incremental lengths of extension for experimentation. Each tendon was stretched by increments of 2% resting length, from 2% to 10%. We did not test to failure. Before testing, the tensometer was zeroed. Five cycles of strain were conducted per incremental increase for each tendon. Tensile strain was increased at a rate of 50 mm min^−1^. The tensometer recorded the load (measured in N, resolution 0.001 N) required to lengthen the tendon and the displacement (in mm, resolution at 0.01 mm) digitally at a sample frequency of 50 Hz, using MesurGauge software. Stress calculations took into consideration the cross-sectional area of the tubercle of insertion. Testing continued until all 60 (6 species, 5 increments, 2 legs per species) tests were completed. Stress, strain and modulus of elasticity values were calculated for each hindlimb for each species. Total load per increment was determined by taking the average of the three highest recorded load values for species/leg combination. We accounted for pre-testing tension by setting the zero-load point at the average of the three lowest values measured for the three last cycle troughs. The first of the five cycles, which we identify as pre-stress cycles, was not included into final load values (though it is included in Figs S1 and S2). Final load values per leg were the result of the averaged peak values of cycles 2, 3 and 4. The load was then averaged between both legs to achieve a final load value per species. Stress and moduli values were calculated using these averaged results. The averaged load and the modulus were plotted against increment of extension.

To assess tensile stress σ and strain ε at each length increment, we used the following equations, respectively:
(4)

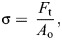
where *F*_t_ equals the applied force to the tendon, and *A*_o_ equals the cross-sectional area of the tendon–bone junction before loading, and:
(5)

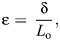
where δ equals the transmitted force length (i.e. the change in length after applied load, or the final length minus the original length), and *L*_o_ equals the original, resting length before loading.

The modulus of elasticity (Young's modulus; *E*) was determined by calculating the slope of the rising stress–strain curves from the hysteresis loops of cycles 2, 3 and 4 at three stages: the toe region, early linear stage and late linear stage, where the slope was greatest. Specifically, the rising curve was divided into three equal regions, and slope of the central five points of each region was computed using the lm function in base R (v. 5.1). The peak slope of stress/strain during the late linear stage was selected for cycles 2–4, averaged for the left and right leg, serving as our primary *E* values for comparison. The areas under the rising and falling stress–strain curves for cycles 2–4 were computed using trapezoidal integration using the trapz function in the pracma R package (v. 2.4.4; https://CRAN.R-project.org/package=pracma). Tendon resilience (percentage of energy recovered) was computed as the area under the falling curve divided by the area under the rising curve. For the focal six species that underwent tensile properties testing, parametric tests were used to assess for significant differences between hawks and owls.

## RESULTS

Morphometric results indicate that strigids have both a lower aspect ratio of the tarsometatarsus and further distally positioned insertion points of the m. tibialis cranialis tendon than the accipitrids, resulting in greater mechanical advantage of the prey lifting lever mechanism. Similarly, across our broader sample size, including extinct taxa, strigids typically showed higher mechanical advantages. Tensile testing of the m. cranialis tibialis tendon resulted in variable values of load, stress and elastic modulus, not significantly different between families. However, strigids did tend to show greater load capacities at similar increments of tendon extension compared with accipitrids. The material properties assessed here resulted in relatively low *E* values, with lower tensile stiffness and greater elasticity compared with terrestrial birds.

### Comparative skeletal results

For the focal species within the heaviest mass category, the length and width of the tarsometatarsus of *B. virginianus* measured 76% and 130% that of *B. jamaicensis*, respectively. The averaged aspect ratio of the tarsometatarsus was approximately 1:7 in *B. virginianus* and 1:11 in *B. jamaicensis*. The proportional distal distance of the m*.* tibialis cranialis tubercle from the proximodorsal margin of the tarsometarsus in *B. virginianus* and *B. jamaicensis* measured 34% and 22%, respectively (Table 1).

**Table 1.** Averaged morphometric measurements for each species

Order	Family	Genus	Species	Tmt length	Tmt width	Tubercle distance	Tubercle to trochlea
Accipitriformes	Accipitridae	*Accipiter*	*striatus*	51.4±3.1	2.6±.46	7.2±.55	44.2±2.77
		*Astur*	*cooperii*	66.91±3.47	4.8±.65	12.66±1.32	54.3±2.53
		*Buteo*	*jamacensis*	82.3±3.2	7.4±.75	18.1±1.3	64.2±2.74
Strigiformes	Strigidae	*Megascops*	*asio*	32.12±2.22	3.49±.35	8.92±.97	68.8±1.37
		*Asio*	*otus*	41.1±1.9	4.5±.36	11.9±1.15	29.1±1.06
		*Bubo*	*virginianus*	62.6±2.22	9.6±.57	21.3±.87	41.3±2.1

All lengths given in mm. Raw measurements can be found in Tables S4 and S5. Tmt, tarsometatarsus.

Within the medium mass category of assessed focal species, the length and width of the tarsometatarsus of *A. otus* measured 61% and 94% that of *A. cooperii*, respectively. The averaged aspect ratio of the tarsometatarsus was approximately 1:9 in *A. otus* and 1:14 in *A. cooperii*. The proportional distal distance of the m*.* tibialis cranialis tubercle from the proximodorsal margin of the tarsometatarsus in *A. otus* and *A. cooperii* measured 29% and 19%, respectively.

For the lightest mass category of assessed focal species, the length and width of the tarsometatarsus of *M. asio* measured 62% and 134% that of *A. striatus*, respectively. The averaged aspect ratio of the tarsometatarsus was approximately 1:10 in *M. asio* and 1:20 in *A. striatus*. The proportional distal distance of the m*.* tibialis cranialis tubercle from the proximodorsal margin of the tarsometarsus in *M. asio* and *A*. *striatus* measured 28% and 14%, respectively.

Overall, aspect ratios and proportional m. tibialis cranialis tubercle distal location between focal accipitrids and strigids were significant (*P*<0.01, *W*=5), as were differences in required muscle force to lift prey based on skeletal metrics between the six focal species (*P*<0.01, *W*=900) (Table 2; Table S1). Metrics of the tarsometatarsus among the broader sample of 66 species, including fossil taxa, show that the hindlimb lifting mechanism varies in significant ways (Table S2). Differences in tarsometatarsus aspect ratios between species of the two families were significant (*P*<0.01, *t*=−4.3, d.f.=56). Similarly, relative distal position of the m. tibialis cranialis tubercle was significantly lower on the tarsometatarsus in strigids compared with accipitrids (*P*<0.01, *t*=−6.09, d.f.=62).

**Table 2.** Averaged biomechanical calculations for each species

Taxa	Prey	RP (N)	RA (m)	FA (m)	MA	Muscle force (N)	Torque (N m)
*Accipiter striatus*	*Tamias striatus* (140 g)	1.37	0.0514	0.0072	0.14	9.8	0.5
*Astur cooperii*		1.37	0.0669	0.0127	0.19	7.23	0.48
*Buteo jamaicensis*		1.37	0.0823	0.0181	0.22	6.24	0.51
*Megascops asio*		1.37	0.0312	0.0089	0.29	4.81	0.15
*Asio otus*		1.37	0.0411	0.0119	0.29	4.74	0.19
*Bubo virginianus*		1.37	0.0626	0.0213	0.34	4.04	0.25
							
*Accipiter striatus*	*Didelphis virginiana* (3600 g)	35.31	0.0514	0.0072	0.14	252.12	12.9
*Astur cooperii*		35.31	0.0669	0.0127	0.19	186.03	12.8
*Buteo jamaicensis*		35.31	0.0823	0.0181	0.22	160.58	13
*Megascops asio*		35.31	0.0312	0.0089	0.29	123.8	3.9
*Asio otus*		35.31	0.0411	0.0119	0.29	121.97	5
*Bubo virginianus*		35.31	0.0626	0.0213	0.34	103.79	6.5
							
*Accipiter striatus*	*Passer domesticus* (34 g)	0.33	0.0514	0.0072	0.14	2.38	0.12
*Astur cooperii*	*Zenaida macroura* (119 g)	1.08	0.0669	0.0127	0.19	6.15	0.73
*Buteo jamaicensis*	*Sciurus carolinensis* (544 g)	5.33	0.0823	0.0181	0.22	24.27	13.2
*Megascops asio*	*Peromyscus arboreus* (20 g)	0.2	0.0312	0.0089	0.29	0.69	0.01
*Asio otus*	*Microtus pennsylvanicus* (43 g)	4.22	0.0411	0.0119	0.29	14.57	0.6
*Bubo virginianus*	*Mephitis mephitis* (3628 g)	35.59	0.0626	0.0213	0.34	104.6	6.55

To assess the required muscle force and torque of a given prey item, we used three different scenarios for each raptorial species: a 140 g *Tamias striatus*, a 3600 g *Didelphis virginiana*, and an upper-end mass estimate of known typical prey items. Calculations reflect values of a single leg. FA, force arm; MA, mechanical advantage; RA, resistance arm; RP, resistance pressure.

Differences in required muscle force to lift prey was significantly different among the broader surveyed species (*P*<0.01, *t*=7.33, d.f.=60) (Table S2). Of the broader species dataset, phylogenetic signal for all morphological and functional traits of extant hawks and owls was high (Pagel's lambda >0.9). Testing for significant differences between hawks and owls using phylogenetically informed ANOVAs showed no significant differences between birds of prey groups across all traits. Similarly, a phylogenetic MANOVA showed no significant differences between hawks and owls in overall morphology and function (Table S3).

**Fig. 5.**
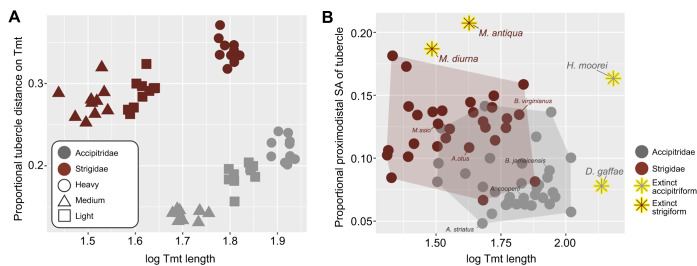
**Variations of the tarsometatarsal morphology. (**A) Proportional distal location of the m. tibialis cranialis tubercle against logged proximodistal length of the tarsometatarsus (Tmt) from three strigid species (10 individuals per species) and three accipitrid species (10 individuals per species) (data from Table S1). (B) The tarsometatarsi of four extinct taxa (two acciptriforms and two strigiforms) compared against a total of 62 extant accipitriforms and strigiforms. SA, surface area.

Extinct strigiform taxa exhibited greater relative lengths of the m. tibialis cranialis tubercle than any other strigiform sampled (Table S2), and yet still yield near-median values for the length of the tarsometatarsus (Fig. 5). The required muscle force of the m. tibialis cranialis to lift ∼3.6 kg among these extinct taxa was within range of that of extant strigiforms of similar size (Table S2). The extinct accipitrids *H*. *moorei* and *D*. *gaffee* also possess the longest tarsometatarsi of any recorded member of this family; however, only *H*. *moorei* exhibits an atypically large surface of the m. tibialis cranialis tubercle relative to the size of the tarsometatarsus (Fig. 5). Similar to the extinct strigiforms, the required muscle force to lift ∼3.6 kg among these extinct accipitrids was within the range of that of extant acciptriforms, even when considering their size (Table S2).

### Mechanical advantage and torque at the ankle joint

The mechanical advantage (MA) of the hindlimb dorsiflexion mechanism was greater among the focal strigids (0.29–0.34) than in the accipitrids (0.14–0.22) (*P*=0.01, *W*=0; Table 2). This trend was also present in the larger survey of accipitrid and strigid limb metrics (*P*<0.01, *t*=−6.09, d.f.=62; Table S2), with the MA for accipitrids ranging from 0.14 to 0.34 (mean=0.21, s.d.=0.05) and that for strigids from 0.2 to 0.41 (mean=0.29, s.d.=0.06). Similarly, for each size category, strigids required less torque to lift prey items of similar size compared with accipitrids (*P*=0.01) (Table 2). The lower aspect ratios of strigid tarsometatarsi, often facilitated by the proportionally shorter length, reduces the outlever arm length (RA), making the ratio of RA and FA closer to 1 compared with accipitrids. An accipitrid with a longer tarsometatarsus would therefore increase the length of the resistant arm (RA), resulting in a greater amount of required torque (*F*). For example, *B*. *virginianus* would only require ∼64% the amount of torque compared with *B. jamaicensis* to lift comparable prey items (Table 2). Furthermore, *A. otus* required ∼66% the amount of torque compared with *A. cooperii* to lift comparable prey items, and *M. asio* required ∼49% the amount of torque compared with *A. striatus* to lift comparable prey items (Table 2). This trend was also present among the larger survey of accipitrid and strigiform metrics (*P*<0.01) (Table S2).

### Tensile material properties of the m. tibialis cranialis tendon

Cyclic tensile testing of the avian m. tibialis cranialis tendon yielded load versus extension raw data plots for each species typical of tendon tensile tests, and produced J-shaped stress–strain hysteresis curves that are useful for computing loading and unloading parameters of the tendons (Figs S1, S2). At the highest strains of 10% elongation, peak stresses among accipitrids ranged from 1.45 to 4.46 MPa, and from 1.6 to 11.19 MPa among strigids. At 10% elongation, the averaged lowest resulting moduli of 17.2±2.8 MPa was found in the strigid *B. virginiaus*, whereas the highest average moduli of 128±9 was presented in the strigid *M. asio* (Table 3; Table S4, Fig. S2).

**Table 3.** Summarized tensile testing measurements and results from six birds of prey at 10% tendon elongation

	*Accipiter striatus*	*Astur cooperii*	*Buteo jamaicensis*	*Megascops asio*	*Asio otus*	*Bubo virginianus*
Body mass (g)	95–200	275–667	680–1600	163–263	204–409	900–1800
Tendon length (mm)	13.12	14.88	19	13.25	17.79	20.25
Tendon stretched length (mm)	14.43	16.38	20.9	14.58	19.59	22.28
Tendon CSA (mm2)	3.85	5.05	17.56	2.3	6.58	19.35
Peak load (N)	10.5±2.89	21.7±2.64	28±2.56	25.6±2.82	24.8±9.72	27.6±2.8
Peak stress (MPa)	2.72±0.53	4.3±0.16	1.59±0.14	10.73±0.46	3.77±0.59	1.425±0.18
Peak modulus (MPa)	34.6±5.4	46±2.6	18.6±2.6	128±9	53.3±7.6	17.2±2.8

Values are given for the resting tendon length and stretched tendon length once at 10%. Peak load (N), stress (MPa) and moduli (MPa) values are given with standard deviations. CSA, cross-sectional area.

For the heaviest mass category, the force (N) required to extend the tendon by 10% beyond its resting length in *B. virginianus* was 80–120% (24.1 N left, 31 N right) that of *B. jamaicensis* (30.1 N left, 25.8 N right). Averaged stress applied at this same extension increment measured 1.43 MPa in *B. virginianus* and 1.79 MPa in *B. jamaicensis* (Tables S4, S5). The averaged modulus of *B. virginianus* measured 92% that of *B. jamaicensis* at 10% elongation.

For the medium mass category, the force required to extend the tendon 10% of its resting length in *A. otus* was 97–132% (28.4 N left, 21.2 N right) that of *A. cooperii* (21.9 N left, 21.5 N right). Averaged stress applied at this same extension increment measured 3.77 MPa in *A. otus* and 4.3 MPa in *A. cooperii*. The averaged modulus of *A. otus* measured 116% that of *A. cooperii* at 10% elongation.

For the lightest mass category, the force required to extend the tendon 10% of its resting length in *M. asio* was 199–307% greater (24.7 N left, 26.4 N right) than that of *A. striatus* (8.6 N left, 12.4 N right). Averaged stress applied at this same extension increment measured 10.73 MPa in *M. asio* and 2.73 MPa in *A. striatus*. The averaged modulus of *M. asio* measured 370% that of *A. striatus* at 10% elongation.

**Fig. 6.**
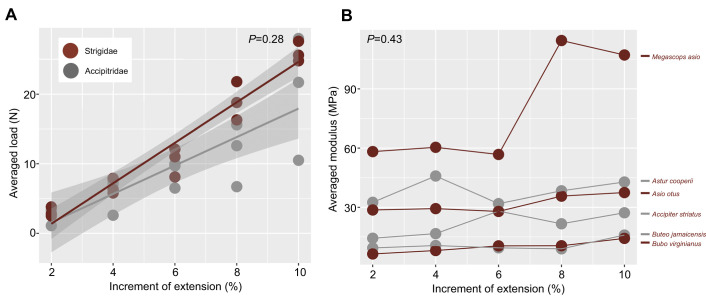
**Load and modulus results.** (A) Averaged tensile load (N) required from each species' increment of elongation and (B) averaged peak modulus (MPa) from each species against strain. Moduli values represented here are from using peak load values from the central three cycles during tensile testing (refer to Table S4).

**Fig. 7.**
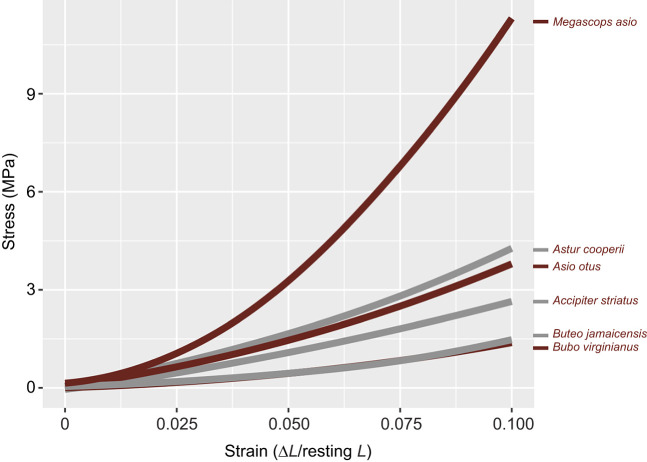
**Stress against strain results.** With the exception of *M. asio*, other sampled taxa tend to closely pair with species within their mass ‘class’.

Averaged load results between accipitrids and strigids per increment of extension resulted in insignificant values (*P*=0.28, *t*=−1.1, d.f.=28), failing to support our hypothesis of strong hawk–owl differences. However, results suggest a greater positive relationship between increment of extension and load required for strigids compared with accipitrids (top three recorded load values per increment test, per species, is included in Table S5) (Fig. 6; Tables S4, S5). Stress at 10% elongation did not significantly vary between families (*P*=0.4, *t*=–0.84, d.f.=4), though *M. asio* did result as an outlier from other focal species (Table S4; Fig. 7). Note that different resulting loads for the left and right tendons of each species may have been influenced by imperfect placement within the tensometer between leg testing or machine error.

Using peak load values from the three central cycles to calculate the averaged peak Young's moduli between accipitrids and strigids per increment of extension did not result in statistically significant *P*-values (*P*=0.43, *t*=–0.99, d.f.=2), though moduli did decrease in the highest mass category species (*B. virginianus*, *B. jamaicensis*) compared with the medium and small categories (Table 3; Tables S4–S6). The resulting moduli suggest a greater positive relationship in strigids relative to accipitrids, suggesting overall decreased comparative tendon elasticity (Fig. 6), though this decreased in the heaviest strigid sampled (*B. virginianus*).

Assessment of hysteresis loops at 10% elongation across sampled species indicated moderate energy loss within the central three cycles of stretching (Table S4, Fig. S3), suggesting that these tendons lost some energy between each testing cycle. The moduli of the rising lines of the hysteresis loops of cycles 2, 3 and 4 resulted in the greatest values in cycle 2 and the lowest in cycle 4 for nearly all sampled taxa (Table S4). Of the three cycles per species, the averaged resiliencies ranged between 0.62 (*A*. *cooperii*) and 0.81 (*M. asio*) (Tables S4–S6, Fig. S3).

## DISCUSSION

To acquire prey, accipitrids and strigids pin down, grasp and lift with the hindlimbs, highlighting the importance of ankle dorsiflexion and the significance of structural and functional variability in this mechanism. The results presented here show that there are significant differences between accipitrid and strigid hindlimb skeletal anatomy, such as the aspect ratio of tarsometatarsi, relative distal position of the m. tibialis cranialis tubercle, required muscle force, and the mechanical advantage of the lifting lever mechanism of the hindlimb. Though skeletal differences are significant, our results show that there are no significant differences between the load-bearing capabilities and elastic moduli between accipitrid and strigid m. tibialis cranialis tendons. Importantly, the elastic moduli in our sample of accipitrids and strigids are significantly lower than those of cursorial birds such as *Meleagris gallopavo*, as initially predicted (peak accipitrid and strigid values measuring only ∼0.04% and ∼0.1%, respectively, that of *M. gallopavo*; Matson et al., 2012). The central conclusions of our findings are fourfold: (1) strigids exhibit much lower aspect ratios of the tarsometatarsus, meaning they are robust, facilitating mediolaterally wider, stronger tendons as has been reported elsewhere (Ward et al., 2002); (2) the distal position of the m. tibialis cranialis insertion significantly differs between accipitrid and strigids, resulting in important changes to hindlimb lever mechanics; (3) tensile properties of the m. tibialis cranialis between accipitrids and strigids are comparable in members of the larger size classes; and (4) morphology of the tarsometatarsi of extinct members of Accipitriformes and Strigiformes are unusual in their relative tubercle size and distal position, suggesting atypical prey acquisition strategies compared with extant relatives. Here, we discuss implications of our results pertaining to extant birds of prey and how these findings can be applied to reconstructing potential hindlimb function in extinct birds of prey.

### Skeletal morphology significantly influences hindlimb biomechanics

In the focal accipitrid species assessed here, the aspect ratio of the tarsometatarsus changes with size, becoming more robust (*A. striatus, A. cooperi* and *B. jamaicensis* exhibited aspect ratios of 1:20, 1:14 and 1:11, respectively) (Table S1). This allometric scaling may signal differences in both prey size and behavior upon capture, and a subsequently greater need to subdue it. Indeed, each focal accipitrid's typical prey species tend to increase in proportional mass and decrease in relative speed (e.g. passerines to medium-sized mammals) (del Hoyo et al., 1999). However, among the focal strigids, tarsometatarsal robustness remains much more conserved, ranging instead from 1:10 to 1:7. In contrast to accipitrids, strigids may implement similar prey grasping strategies and experience similar levels of proportional resistance from their respective prey species.

Not only do strigid tarsometatarsi have a lower aspect ratio compared with accipitrids, but the insertion for the m*.* tibialis cranialis is nearly always more distally positioned when accounting for body size, providing a longer inlever for the cranialis tibialis muscle. To note, strigids also tend to have more medially located m*.* tibialis cranialis tubercles, which also suggests greater pedal inversion upon contraction (Clark et al., 2024). The variation in location of the corresponding tubercle is therefore an ecomorphologically rich feature, signaling potential grasping capabilities and dorsiflexion force (Moreno and Carrascal, 1993; Zeffer and Norberg, 2003).

Indeed, relative position of this tubercle may signal varying levels of emphasis on dorsiflexion of the ankle *in vivo*. The tubercle appears most proximal in terrestrial birds (e.g. *Struthio*), which require little dorsiflexion of the ankle as their resistance is merely air (Zeffer and Norberg, 2003). Arboreal birds show greater relative distal migration, as dorsiflexing the ankle both helps secure the pes wrapping around branches and brings the center of gravity towards the perch, thus increasing stability (Zeffer and Norberg, 2003). Of even greater relative distance are aquatic birds, which require more potential force to bring the feet upwards against the resistance of fluids (i.e. paddling) (Zeffer and Norberg, 2003).

Among all extant birds, those that are raptorial (e.g. strigiforms, falconiforms, accipitriforms) exhibit the distal-most positioned tubercles, with maximums reaching nearly 45% distally along the length of the tarsometatarsus as typified in the strigid *Surnia ulula*. Therefore, strigids, even when considering our broader sample, exhibit a greater morphological advantage in dorsiflexing the ankle as the more distal position of the tubercle results in a lower required output torque (i.e. muscular force times the outlever length) to either bring their body weight on top of prey or to lift them during flight (Table S2). Perhaps the greatest exemplar of this principle are the diminutive pygmy owls (genus *Glaucidium*), which can subdue and travel with prey items that rival, and often exceed, their own body mass (e.g. *Microtus* sp., *Coccothraustes* sp., *Callipepla* sp*.*) (del Hoyo et al., 1999; Zeffer and Norberg, 2003).

### Tensile properties suggest similar resistances despite foraging differences

Between their mass categories, strigids typically required greater force to elongate the tendon of the m*.* tibialis cranialis compared with accipitrids (Table 2; Table S4). Note that the greater resulting load values in strigids may be influenced by the dual tendinous heads of the m*.* tibialis cranialis (m*.* tibialis cranialis tibial head and femoral head), which both insert onto the tubercle (Volkov, 2004). Additionally, material properties of the m*.* tibialis cranialis tendon emphasize, on average, greater tensile strength and a decreased modulus (i.e. increased elasticity) among larger-bodied owls, perhaps reflecting a need for greater elasticity with an increase in prey size, which produces greater resistance (i.e. potential contortion) upon capture.

Similarly, variations of elasticity (Young's moduli) among accipitrids suggest disparate methods of prey acquisition within the family. Like strigids, the largest accipitrid sampled (*B. jamaicensis*) has the lowest Young's modulus values, indicating possibly similar ecomorphological principles related to prey size and resistance to capture. More diminutive hawks, such as *A. striatus* and *A. cooperi*, tend to capture quick-moving, lightweight birds on the wing, which would require stiffer tendons and likely result in less deformation upon capture (del Hoyo et al., 1999).

However, overall, tensile properties of the m. tibialis cranialis tendon surprisingly showed little variation between nearly all focal accipitrids and strigids with the exception of *M. asio* (Figs 6, 7). Given the differences in foot morphologies, prey acquisition strategies and methods of prey termination (e.g. primarily use of pes versus bill), these results are initially puzzling. Fascinatingly, although soft tissue tensile properties may be similar across the sampled focal species, these structures functioning in tandem with the underlying variations of the skeletal structure facilitate significant differences in hindlimb function concerning catching, subduing and transporting prey. The interesting outlier is *M*. *asio*, which yielded approximately 3.5 to 4 times the amount of the stress and resulting moduli values of *A*. *striatus* (same mass category), respectively – much higher than other mass category pairings (Table 3). These results suggest that *M. asio*, though the smallest strigid sampled, has the most rigid and unyielding m. tibilias cranialis tendon of any species assessed – which may uniquely be associated with preferred prey items (Abbruzzese and Ritchison, 1997). Though both *A*. *striatus* and *M*. *asio* often forage for prey of similar size (e.g. *Melospiza* spp., *Mus* spp., ∼20 g), nearly 50% of the diet of *M*. *asio* can be crayfish (*Cambarus* spp*.*), which are often twice the mass of the other organisms that constitute their diet (Abbruzzese and Ritchison, 1997). Though the tendons of *M*. *asio* are smaller concerning their absolute size than those of other raptorial birds, they are proportionally much stiffer in tension. This proportional disparity may be due to the relative tremendous density of the outer integument of their prey that they must overcome to pin or maintain grasp of. Additionally, if these prey items are being transported, then *M*. *asio* are carrying approximately twice the amount of proportional weight as the other light-class raptorial species (*A*. *striatus*).

In previous studies assessing similar questions in mammals, nearly all sampled species above 40 MPa of stress were able to yield moduli values of over 1 GPa (Bennett et al., 1986; Mossor et al., 2020; Pollock and Shadwick, 1994). In our focal raptorial birds, the ranges of moduli in the m. tibialis cranialis tendon ranged from approximately 17.2 to 128 MPa. These significantly differ from mammals and terrestrial birds (e.g. *M. gallopavo*), whose values can exceed 1 GPa under similar stresses (Matson et al., 2012; Pollock and Shadwick, 1994). However, unlike these prior studies, we did not test to failure but instead to a maximum increment of an additional 10% of resting length. Supporting our initial hypothesis, these arboreal raptorial birds exhibit much lower values of this tendon's tensile properties. This is likely attributable to two key ecological differences, the first of which is that birds of prey do not primarily locomote terrestrially. Unlike most mammals and *M. gallopavo*, raptorial birds require comparatively more elastic tendons to grasp and grapple with prey items upon capture, perhaps with the exception of *M. asio*. This also relates to the second major difference: the m. tibialis cranialis tendon is significantly utilized only during temporary conditions as pinning or lifting prey items. This is in contrast to repetitive, cyclical movements such as the cursorial locomotion of turkeys (e.g. walking and running). We suggest that the more elastic tendons of the raptors studied here may be functioning in shock absorption during the initial prey capture event, and may play a role in retaining a flexible grip on struggling prey during lifting and transport. These results highlight that among organisms, tendon moduli will vary depending upon ecological context.

### Implications for extinct taxa

Understanding the ecological implications of extant raptorial tarsometatarsi morphology can prove useful for predicting how extinct birds of prey may have implemented their hindlimbs during prey capture. The assessed extinct strigiforms, *Minerva antiqua* and *Miosurnia diurna*, are both predicted to forage similarly to modern strigids (Kurochkin and Dyke, 2011; Li et al., 2022; Mourer-Chauvire, 1983). The robust pedal digit morphologies of *M. antiqua* led the authors to suggest a similar raptorial ecology as the extant members of *Bubo*, albeit with possibly more powerful pedal grip facilitated by short proximal phalanges of pedal digits I and II (Mourer-Chauvire, 1983). *Miosurnia diurna* is thought to be a close cousin to extant members of the genus *Surnia*, and based on sclerotic ring morphology, was similarly diurnal (Li et al., 2022). Weakly grooved trochlea of metatarsal III of *M. diurna* suggest a possible weaker grip than extant members of *Surnia* (Li et al., 2022). Interestingly, although the two extinct strigiforms have typical tarsometatarsus lengths compared with extant species, they have the largest proportional m. tibialis cranialis tubercles. Particularly, the proportionally large size of the m. tibialis cranialis tubercle of *M. antiqua* suggests high stresses at the tendon–bone junction, which may in turn signal relatively powerful muscle contractions (Mourer-Chauvire, 1983).

*Dynatoaetus gaffae* and *H. moorei* represent the two largest accipitriforms ever described. The largest extant accipitrid, *Harpia harpyja*, can weigh up to approximately 9 kg (Billerman et al., 2022). *Dynatoaetus gaffae* is estimated to have weighed up to 11.8 kg, and *H. moorei* perhaps up to 18 kg (Knapp et al., 2019; Mather et al., 2023). *Dynatoaetus gaffae* is hypothesized to have attacked live prey, similar to extant accipitrids, utilizing the pedal digits and talons to facilitate capture and terminating prey via the bill (Mather et al., 2023). Its size is only exceeded by *H. moorei*, most likely due to *D*. *gaffae* experiencing the pressures of living on a continent and having to compete with other carnivores (e.g. *Thylacolea* and varanids), as well as nest predation (Mather et al., 2023). These two pressures were likely absent or far less present for the island-dwelling *H. moorei*, which are known to have preyed upon the massive *Dinornis robustus* (moa), which could have weighed nearly 226 kg (Knapp et al., 2019; Rawlence, 2010). Indeed, the probable cathartid-like feeding behavior of *H. moorei* reflects a foraging strategy that entails subduing proportionally large prey and consuming the carcass over time (Van Heteren et al., 2021).

*Dynatoaetus gaffae* exhibits the second largest tarsometatarsus of any described accipitrid thus far, and yet, the proportional size of the m. tibialis cranialis tubercle is similar to accipitrids nearly half its size (e.g. *Accipiter* sp., *Astur* sp., *Buteo* sp.) (Fig. 5). This suggests relatively high tendon stress, and that the dorsiflexion capabilities of *D. gaffae* were similar to those of extant smaller accipitrids. This may in turn indicate that it either did not often transport prey, or typically foraged for small prey relative to its body size.

*Hieraaetus moorei* exhibits the largest tarsometatarus, and the relatively longest and most distally positioned m. tibialis cranialis tubercle of any known accipitrid (the accipitriform *Pandion* exceeds by a 2% proportional distance; Table S2). Biomechanically, these features together suggest both lever efficiency and immense potential muscle inforce while dorsiflexing the ankle. Interestingly, the extant *Aquila*, *Elanoides* and *Haliaeetus* all require less muscle force to lift the same amount of hypothetical weight (Table S2), but these results may be confounded by the actual physical limits of the birds sampled. Though these birds biomechanically require less muscle force than the larger *H. moorei* to lift prey items, it may be the case they lack the actual muscle mass and subsequent strength to do so. The larger tarsometatarsus and tubercle morphologies suggests that *H. moorei* would likely have been able to apply even greater strength in dorsiflexing the ankle than those extant accipitrids. The large muscle insertion surface and distal position are likely adaptations for bringing the body closer to the legs (i.e. dorsiflexing to fold the legs) to maintain prolonged and strong grip onto larger prey items (e.g. *D*. *robustus*). By maintaining a sustained lower center of gravity on top of their prey while further driving in their talons, *H. moorei* may have had a unique foraging strategy to subdue the proportionally largest prey items of any accipitrid in Earth's history (Rawlence, 2010).

## Supplementary Material

10.1242/jexbio.251052_sup1 Supplementary information

Table S1. All individuals and measurements included in tarsometatarsus comparative analyses

Table S2. Data for extant and extinct taxon tarsometatarsal comparisons

Table S3. Results of phylogenetically informed tests of extended sample

Table S4. Hysteresis loop and moduli data. For each species tested, and for each leg at 10% increment of extension, we assessed the central 3 hysteresis loops (of five total cycles). Of these three cycles, we calculated the slope of the rising (loading) at three evenly-spaced sections, dubbed as the starting, middle, and end slopes. Units for the moduli of these three rising curve sections are in MPa. Additionally, total area within each hysteresis loop and the difference in area under each curve (rising and settling), per cycle were calculated to demonstrate energy loss. Resulting moduli are the result of the averaged 6 end slopes (3 end slopes per leg tested).

Table S5. Results of m. *tibialis cranialis* tendon mechanical testing of six species

Table S6. The top three maximum load values per foot (left or right), per increment (2% - 10%) for each species.
