# Supplementary material for: Biomechanics of raptorial dorsiflexion and tensile material properties of the m. tibialis cranialis tendon in the hindlimbs of hawks and owls
Source: J Exp Biol. 2025 Dec 10;228(23):jeb251052. doi: 10.1242/jeb.251052 (PMC12746076; doi:10.1242/jeb.251052)
Supplement: Supplementary information [file jexbio-228-251052-s1.pdf]

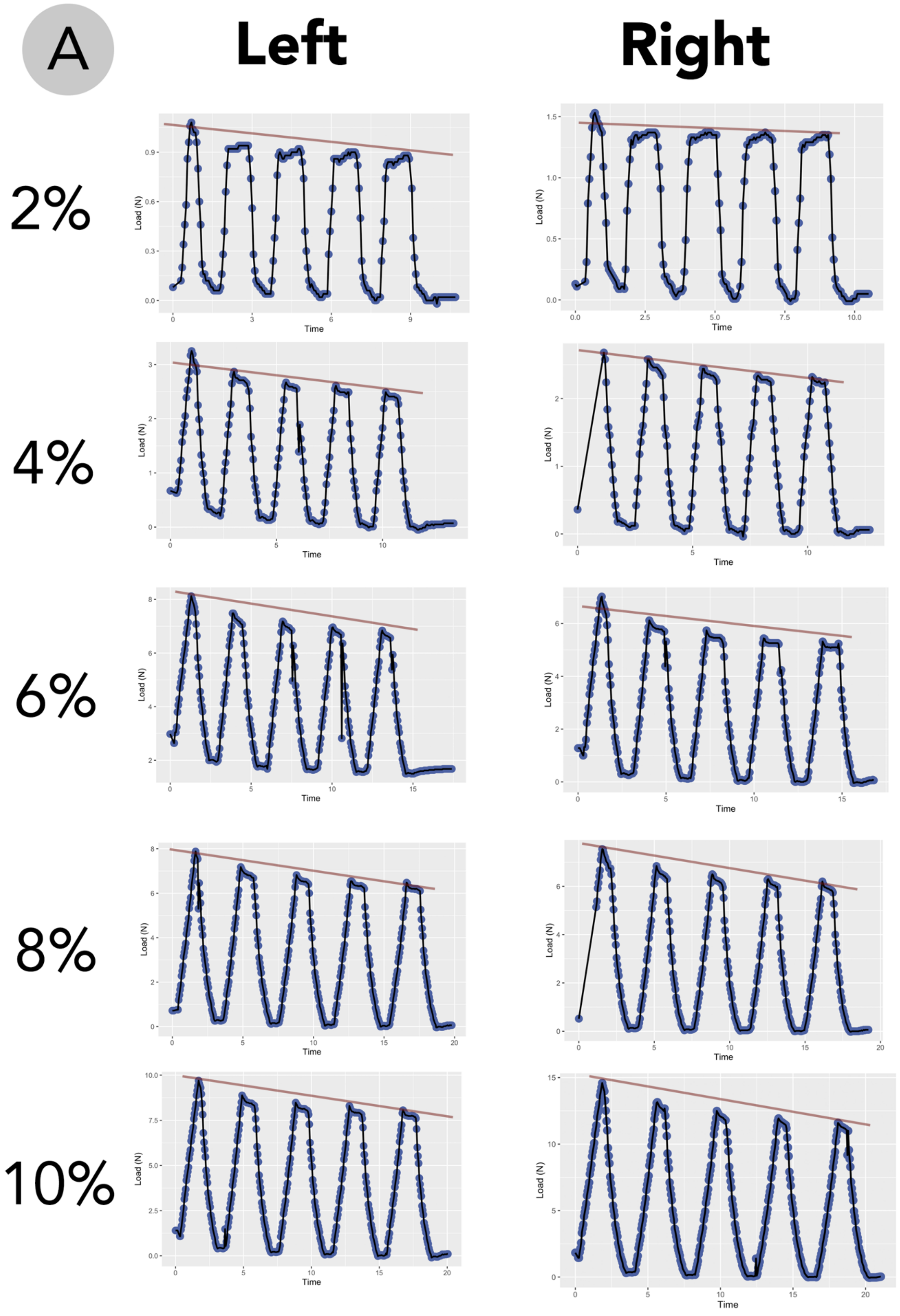

Journal of Experimental Biology • Supplementary information

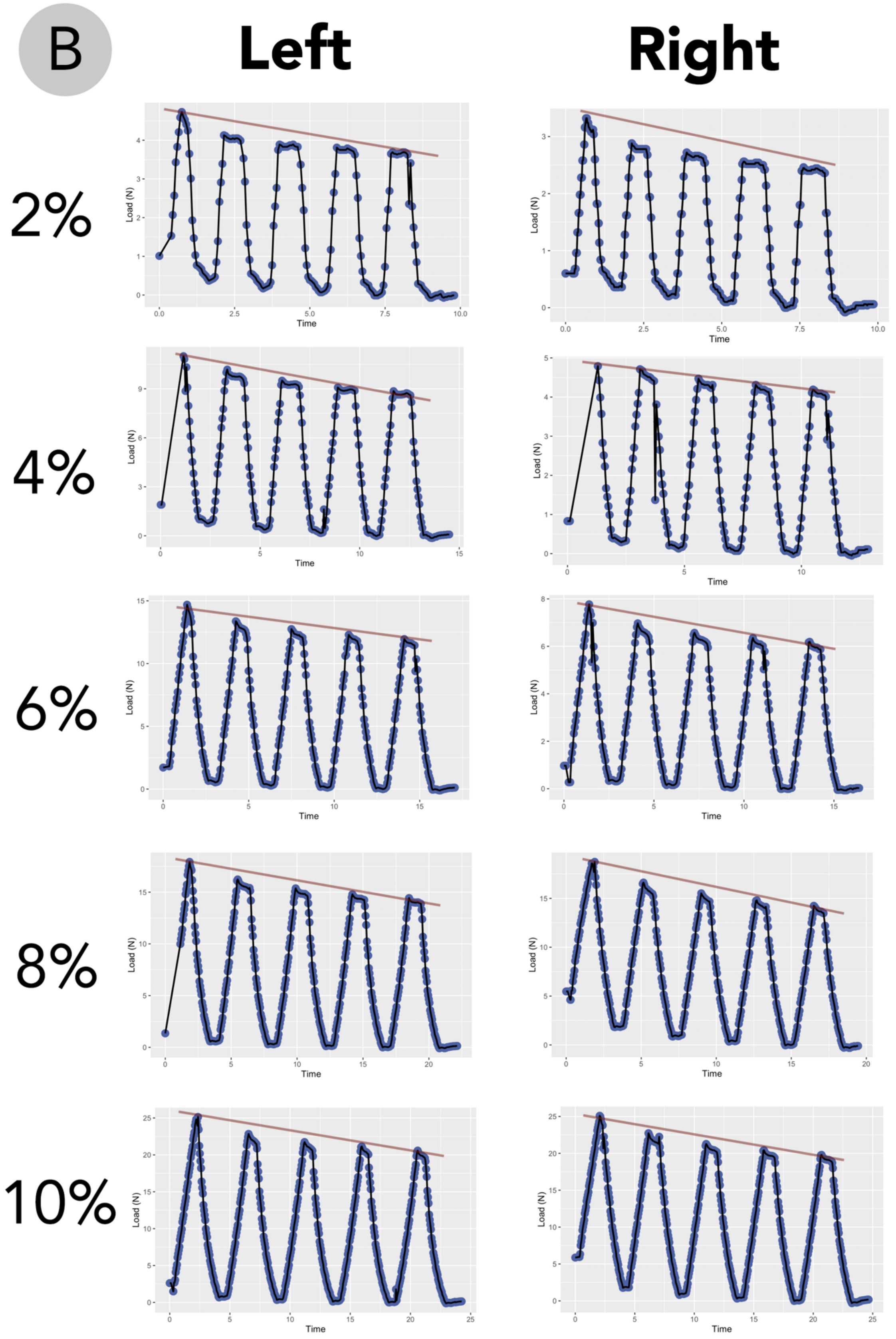

Journal of Experimental Biology • Supplementary information

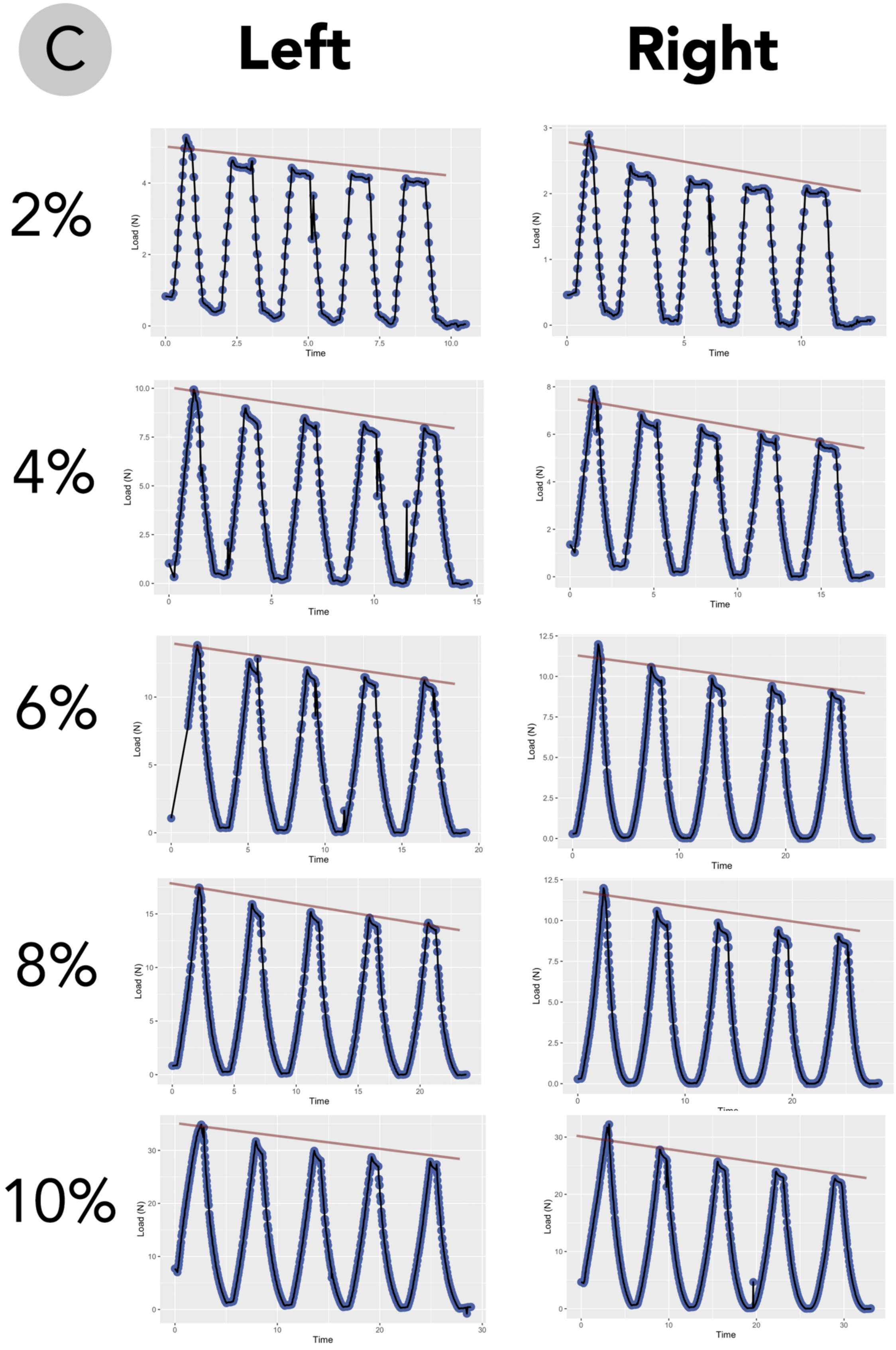
C
Left
Right
2%
4%
6%
8%
10%
Load (N)
Time

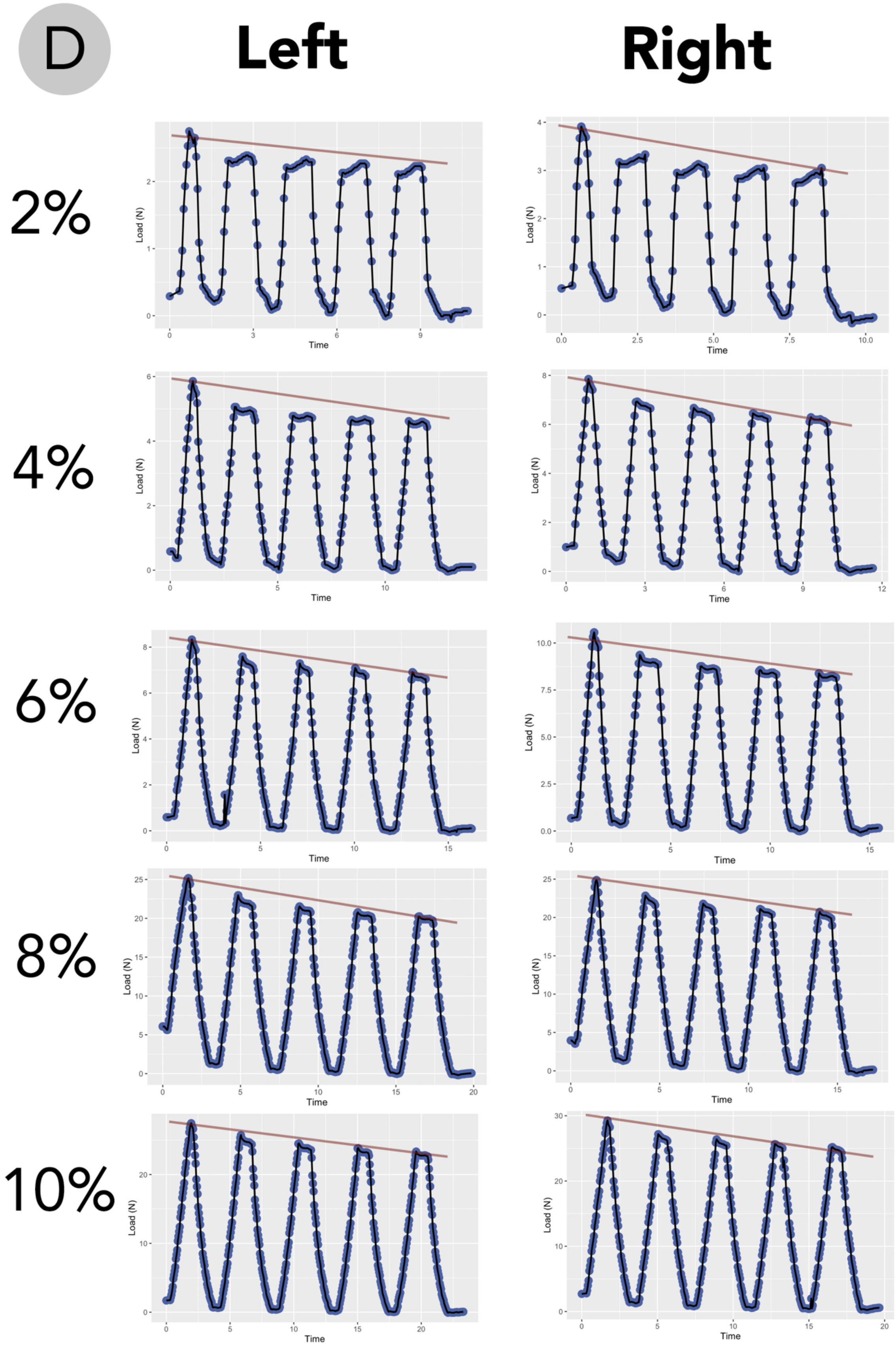
D
Left
Right
2%
4%
6%
8%
10%
Load (N)
Time

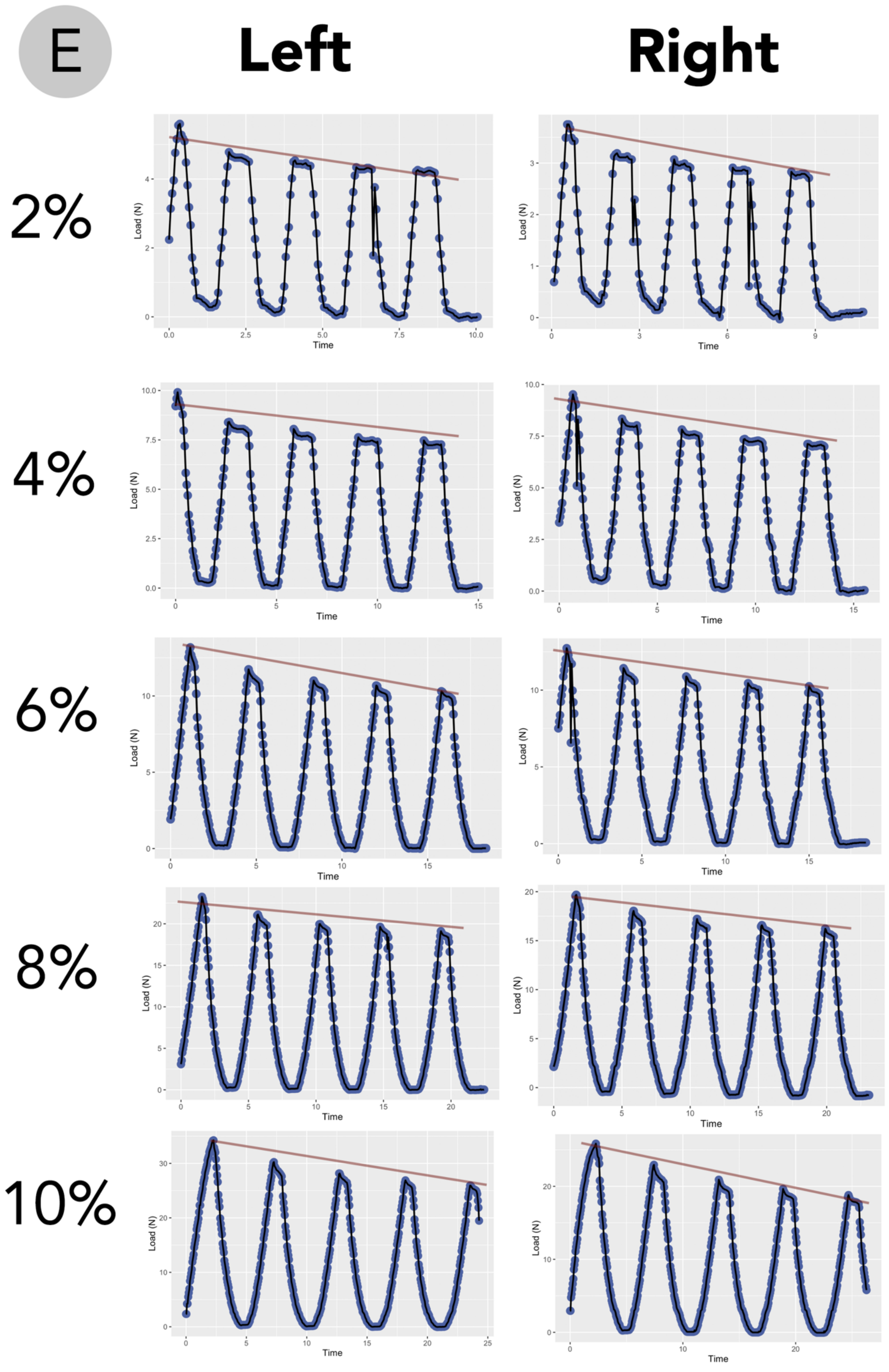

Journal of Experimental Biology • Supplementary information

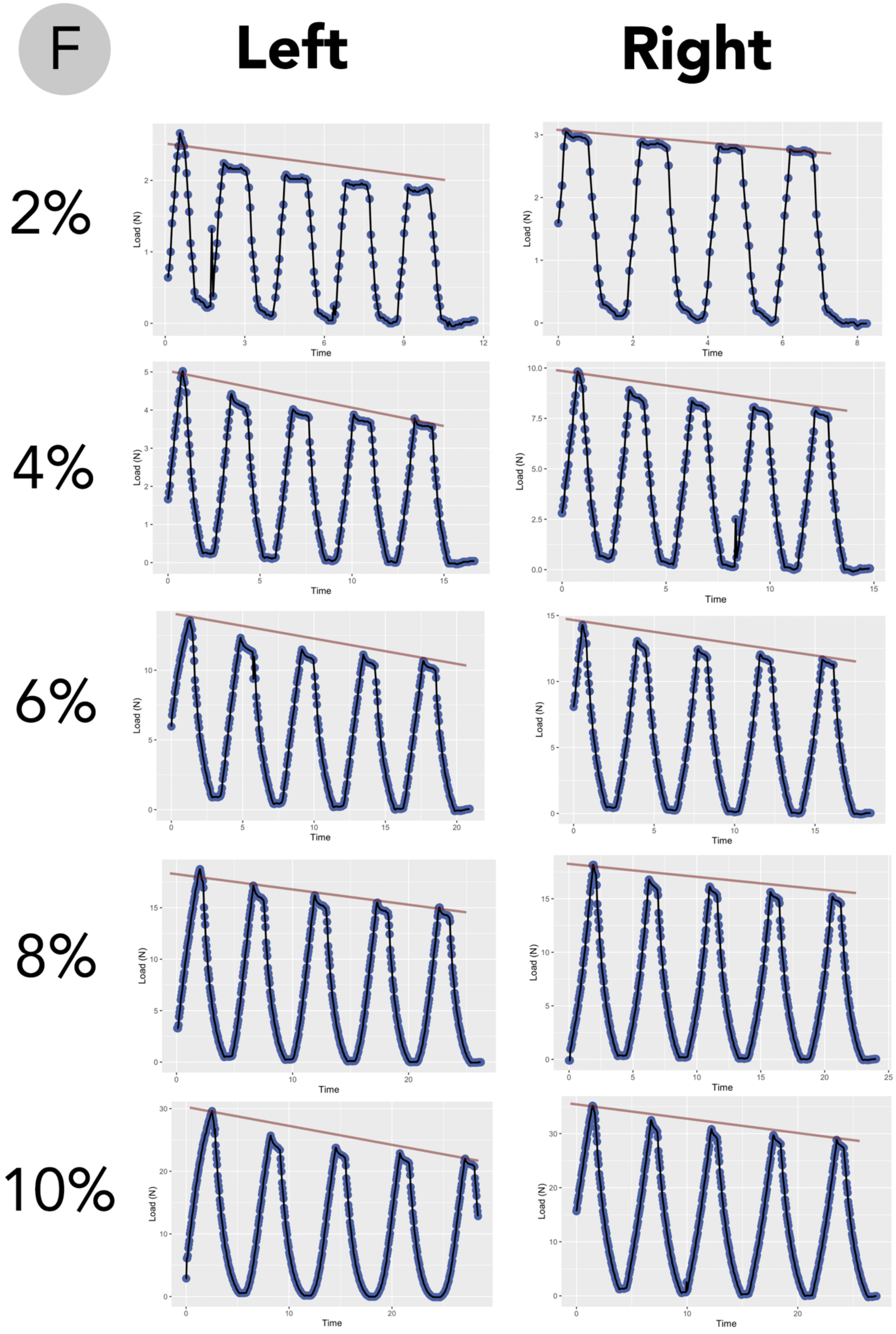

Journal of Experimental Biology • Supplementary information

Fig. S1. Species results for incremental stretching against time over five cycles. A) *Accipiter striatus*, B) *Astur cooperii*, C) *Buteo jamaicensis*, strigids D) *Megascops asio*, E) *Asio otus*, F) *Bubo virginianus*. Red lines approximate peaks of each cycle to visually represent natural stretch of the tendon overtime. Sudden outlier values within the raw cycle data represented machine errors and were removed for the resulting figures.

Journal of Experimental Biology • Supplementary information

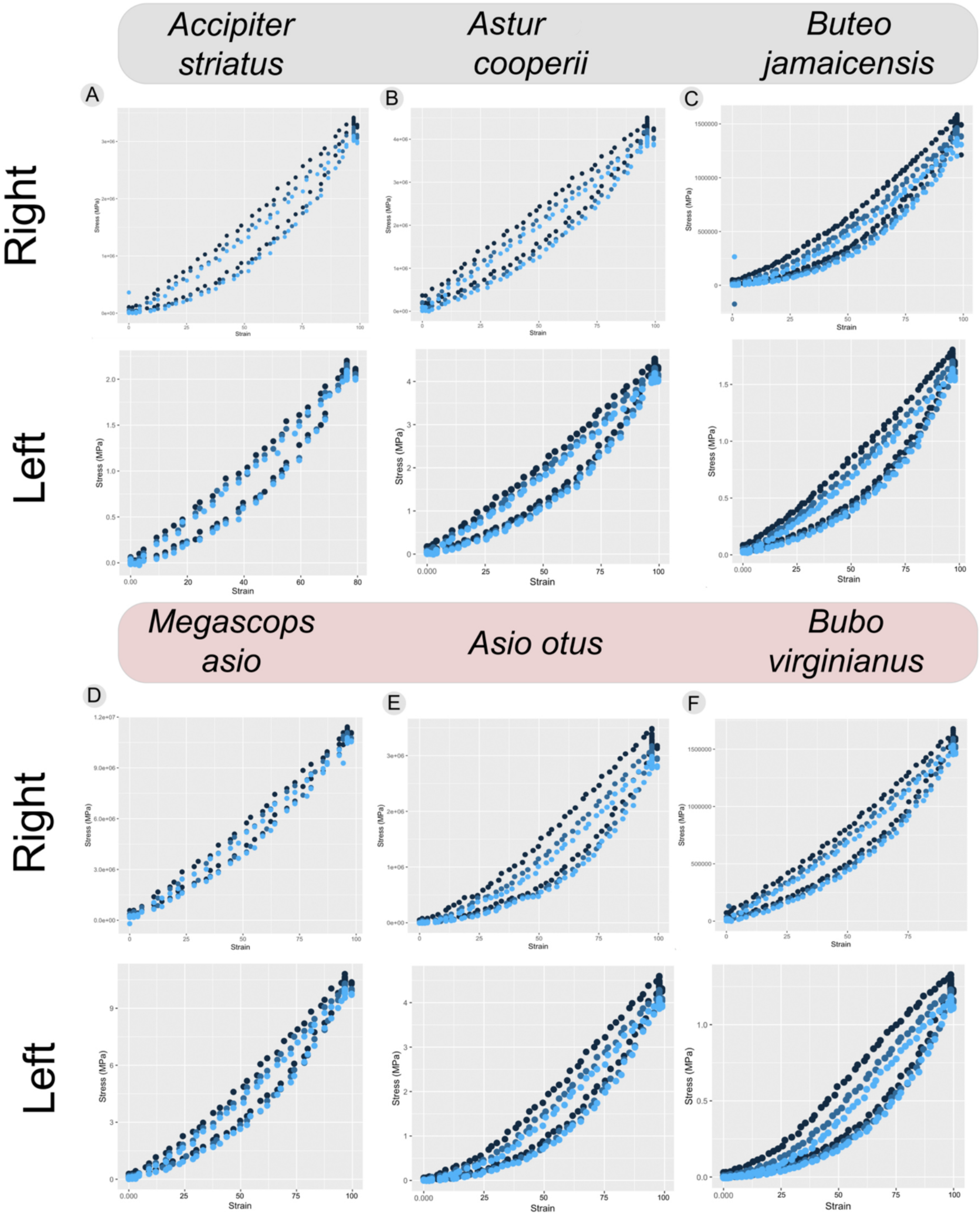

Fig. S2. Hysteresis loops of cycles 2-4 at 10% elongation. A) *Accipiter striatus*, B) *Astur cooperii*, C) *Buteo jamaicensis*, strigids D) *Megascops asio*, E) *Asio otus*, F) *Bubo virginianus*. Cycle 2 is represented by the darkest blue, cycle 3 lighter blue, and cycle 4 the lightest blue. Slopes of the rising curves and loop resiliencies presented in Table S3.

Journal of Experimental Biology • Supplementary information

Table S1. All individuals and measurements included in tarsometatarsus comparative analyses

Available for download at
https://journals.biologists.com/jeb/article-lookup/doi/10.1242/jeb.251052#supplementary-data

Table S2. Data for extant and extinct taxon tarsometatarsal comparisons

Available for download at
https://journals.biologists.com/jeb/article-lookup/doi/10.1242/jeb.251052#supplementary-data

Table S3. Results of phylogenetically informed tests of extended sample

Available for download at
https://journals.biologists.com/jeb/article-lookup/doi/10.1242/jeb.251052#supplementary-data

Table S4. Hysteresis loop and moduli data. For each species tested, and for each leg at 10% increment of extension, we assessed the central 3 hysteresis loops (of five total cycles). Of these three cycles, we calculated the slope of the rising (loading) at three evenly-spaced sections, dubbed as the starting, middle, and end slopes. Units for the moduli of these three rising curve sections are in MPa. Additionally, total area within each hysteresis loop and the difference in area under each curve (rising and settling), per cycle were calculated to demonstrate energy loss. Resulting moduli are the result of the averaged 6 end slopes (3 end slopes per leg tested).

Available for download at
https://journals.biologists.com/jeb/article-lookup/doi/10.1242/jeb.251052#supplementary-data

Table S5. Results of m. *tibialis cranialis* tendon mechanical testing of six species

Available for download at
https://journals.biologists.com/jeb/article-lookup/doi/10.1242/jeb.251052#supplementary-data

Table S6. The top three maximum load values per foot (left or right), per increment (2% - 10%) for each species.

Available for download at
https://journals.biologists.com/jeb/article-lookup/doi/10.1242/jeb.251052#supplementary-data

Journal of Experimental Biology • Supplementary information
